# Detecting clinically relevant rivaroxaban or dabigatran levels by routine coagulation tests or thromboelastography in a cohort of patients with atrial fibrillation

**DOI:** 10.1186/s12959-017-0160-2

**Published:** 2018-02-01

**Authors:** Yvonne M. C. Henskens, Anouk J. W. Gulpen, René van Oerle, Rick Wetzels, Paul Verhezen, Henri Spronk, Simon Schalla, Harry J. Crijns, Hugo ten Cate, Arina ten Cate-Hoek

**Affiliations:** 1 Central Diagnostic Laboratory, Maastricht University Medical Centre (MUMC+), Maastricht, The Netherlands; 2 Laboratory for Clinical Thrombosis and Hemostasis, Internal medicine, CARIM, Maastricht, The Netherlands; 3 Department of Cardiology, Cardiovascular center MUMC+, Maastricht, The Netherlands; 4 Internal medicine, MUMC+, Maastricht, The Netherlands; 5 Thrombosis Expertise Centre, Vascular medicine, Cardiovascular Centre MUMC+, Maastricht, The Netherlands

**Keywords:** Dabigatran, Rivaroxaban, Routine coagulation tests, Pt, aPTT, Tt, ROTEM®, Drug concentration (diltPT, antiXa activity)

## Abstract

**Background:**

Traditional coagulation tests are included in emergency guidelines for management of patients on direct oral anticoagulants (DOACs) who experience acute bleeding or require surgery. We determined the ability of traditional coagulation tests and fast whole blood thromboelastography (ROTEM®) to screen for anticoagulation activity of dabigatran and rivaroxaban as low as 30 ng/mL.

**Methods:**

One hundred eighty-four citrated blood samples (75 dabigatran, 109 rivaroxaban) were collected from patients with non-valvular atrial fibrillation (NVAF), to perform screening tests from different manufacturers, (diluted, D) PT, aPTT, TT and ROTEM®. The activity of DOACs was quantitatively determined by clot detection assays: Hemoclot DTT and DiXaI test (Biophen), on CS2100 (Siemens). The clotting time (CT) of INTEM and EXTEM ROTEM® (Werfen) were used as test parameters.

**Results:**

Dabigatran, ≥ 30 ng/mL, was accurately detected by five coagulation tests: APTT Actin FSL (93%), PT Neoplastin (93%), APTT Cephascreen, Thromboclotin, and Thrombin (all 100%), but not by PT Innovin (49%). CT-EXTEM (91%) was sufficiently sensitive, but not CT-INTEM (52%)*.* APTT Cephascreen and Thrombin showed good linearity (R^2^ = 0.71,R^2^ = 0.72). For the other tests linearity was moderate to poor. Rivaroxaban was accurately detected by PT Neoplastin (98%) and less so by APTT Cephascreen (85%). In addition, rivaroxaban was also accurately detected by CT-INTEM (96%). PT Neoplastin showed good linearity (R^2^ = 0.81), all other tests had moderate to poor linearity.

**Conclusion:**

In patients with NVAF, the ability of routine coagulation tests to detect the presence of significant levels of DOACs is test and reagent dependent. CT-INTEM and CT-EXTEM may be fast whole blood alternatives.

**Trial registration:**

The Institutional Review Board of the MUMC approved this study (December 2011, project number 114069).

## Background

For more than 60 years vitamin K antagonists (VKA) were the drugs of choice to prevent thrombosis in patients with NVAF and mechanical heart valves, as well as to treat and prevent recurrence of thrombosis in patients with venous thromboembolism (VTE).

The management of patients on anticoagulant therapy was guided by laboratory testing of prothrombin time (PT), which was internationally harmonized by the ISI factor resulting in (international normalized ratio) INR, an easily interpretable laboratory test [1]. With the introduction of direct oral anticoagulants it was emphasized that no routine laboratory testing would be needed [2–7]. However, in emergency situations the need for testing remains since residual activity of the anticoagulant treatment might introduce e.g. bleeding during surgery. Screening tests for detecting anticoagulants, which can be performed 24 h a day instead of just during working hours should therefore be available to guide treatment decisions. The concentration below 30 ng/mL is proposed as a safe-for surgical treatment threshold [8]. Thereby, this concentration is sufficient to administer antidote against DOAC if needed [9].

Activity-based drug levels in plasma can be determined but are not obtainable in all care settings [10]. Traditional routine coagulation tests are widely available and may be used for first line testing in emergency situations [11–15]. In many countries regional guidelines have been issued upon the introduction of the DOACs indicating the preferred strategies of action for both the prescription and (laboratory) management of these drugs [16–18]. These guidelines do indicate that the thrombin time (TT) is the most sensitive routine coagulation assay for detection of dabigatran and that the PT is the most sensitive routine coagulation assay for detection of rivaroxaban. Guidelines do however not (sufficiently) report on the sensitivity of the different individual TT, PT and activated partial thromboplastin Time (aPTT) tests that are commercially available. This may be due to the fact that most of these guidelines were based on laboratory data using plasma spiked with DOAC instead of samples acquired from patients using the drugs.

We therefore set out to evaluate two proposed screening algorithms [16, 18] and at the same time validate seven different routine anticoagulation tests for screening of plasma drug activity in daily practice, using plasma samples from a cohort of patients on dabigatran and rivaroxaban. In addition we evaluated ROTEM® for the same purpose in whole blood in a subsample of the study population [19].

## Methods

### Patients

This analysis comprises the first 76 non-valvular Atrial Fibrillation (NVAF) patients (30 on dabigatran, 46 on rivaroxaban) that were structurally followed in an observational DOAC study in the Maastricht University Medical Center (MUMC+), the Netherlands. The study is ongoing.

### Study population

All patients came from a single institution, the anticoagulation clinic Maastricht, and were referred by cardiologists of the MUMC. Patients were followed in an observational cohort study, which started January 2012 and is still ongoing. The Institutional Review Board of the MUMC approved the study (December 2011, project number 114069). All patients enrolled in the follow-up signed informed consent. All consecutive patients with NVAF and a CHADS_2_ score ≥ 2 who were initiated on a DOAC were eligible for inclusion in the study.

### Study design

This laboratory study is part of a prospective observational cohort study. Patients were invited to a special structured nurse-based office visit at the anticoagulation clinic. All patients were followed for 1 year, during which 5 visits were planned (at start of DOAC therapy, 1 month after start DOAC, 3 months, 6 months and 12 months). Important parameters recorded during these visits were: thrombotic events and bleeding complications, side effects of medication, compliance to medication, intermittent illness and/or hospital admissions and renal function. Renal function was assessed at 4 points in time; additional blood for testing of a panel of laboratory tests was drawn. The panel of laboratory tests consisted of: APTT Actin FSL (Siemens), APTT Cephascreen (Stago), PT Innovin (Siemens), PT neoplastin (Stago), Diluted PT (DPT) Innovin (Siemens), TT Thromboclotin (Siemens), TT Thrombin (Stago), CT INTEM (ROTEM®), and CT EXTEM (ROTEM®, Werfen). Diluted TT Hemoclot (Biophen) was set as gold standard for the detection of dabigatran and anti Xa activity DiXal (Biophen) for the detection of rivaroxaban. For this paper clinical details are not presented because the blood samples were purely used for laboratory validation.

### Blood sampling and laboratory measurements

Blood was taken by antecubital venipuncture and collected into 3,2% citrated vacutainer® tubes using a 21 gauche eclipse signal blood collection needle (BD vacutainer®), sampling was standardized and timed between 9 and 11 a.m.

Plasma was obtained after centrifugation for 10 min at 2000 g at room temperature. Plasma samples were frozen at −80 °C and thawed in a standard procedure before coagulation testing. PT and DPT Innovin, APTT Actin FSL, TT Thromboclotin and diluted TT Hemoclot as well as Anti-Xa activity were performed on a CS2100 analyzer (Siemens). PT Neoplastin, APTT Cephascreen, TT Thrombin were performed on a STA-R analyzer (Stago), Whole blood was used for ROTEM® analyses. Table 1 summarizes the different methods. The cut-off for a clinical relevant detection limit was set at ≥30 ng/mL for both rivaroxaban and dabigatran as determined as anticoagulation activity by the diluted TT Hemoclot and DiXaI test (both Biophen) on CS2100 using dabigatran and rivaroxaban as calibrators.

**Table 1:** Overview of screening tests, analysers and cut-off values used in this study

	Reagent	Analyser	Firm	Cutt-off
PT	Innovin	CS2100	Siemens	12 s
PT	Neoplastin	STA-R	Stago	15 s
aPTT	Actin FSL	CS2100	Siemens	32 s
aPTT	Cephascreen	STA-R	Stago	32 s
TT	Thromboclotin	CS2100	Siemens	25 s
TT	Thrombin	STA-R	Stago	25 s
CT	INTEM (Tem)	ROTEM	Siemens	195 s
CT	EXTEM (Tem)	ROTEM	Stago	60 s
Diluted TT	Hemoclot	CS2100	Hyphen	30 ng/mL
Anti-Xa activity	DiXal	CS2100	Hyphen	30 ng/mL

*APTT* Activated partial thrombin time, *PT* Phrothrombin time, *TT* Thrombin time, *CT* Closure time, *Sec* Seconds

### Statistics

The statistical package of Graph Pad® (version 6) was used for statistical analysis. Correlations were analyzed by linear regression analysis and slope, intercept and R^2^ were determined and presented. For the sensitivity the cut-off for detection for all routine laboratory tests was set at 30 ng/mL for both dabigatran and rivaroxaban. Sensitivity and specificity were calculated using a diagnostic test evaluation calculator (MedCalc®).

## Results

In total 184 citrated blood samples (75 dabigatran samples and 109 rivaroxaban samples) were collected. Dabigatran activity in a steady state situation, determined by diluted TT Hemoclot varied considerably between patients: Mean _geometric_ 104 (± 53) ng/mL. Rivaroxaban activity determined by DiXaI test showed similar variability, mean _geometric_ 187 (± 139) ng/mL. Clinical relevant dabigatran activity (≥ 30 ng/mL) was detected accurately by both thrombin time assays: TT Thromboclotin (100%), and TT Thrombin (100%). In addition good sensitivity was reached for four other coagulation tests in the panel: APTT Actin FSL (93%), APTT Cephascreen (100%), PT Neoplastin (93%), but not for PT Innovin (49%). Also CT-EXTEM of ROTEM® (91%) was sufficiently sensitive, but not CT-INTEM (52%). (Table 2).

**Table 2:** Sensitivity and specificity, PPV and NPV of a panel of coagulation tests for detecting > = 30 ng/mL dabigatran or rivaroxaban and correlation with the calibrated activity measurement of dabigatran or rivaroxaban

	Dabigatran					Rivaroxaban				
Detection ≥30 ng/mL	Sensitivity	Specificity	PPV	NPV	R^2^	Sensitivity	Specificity	PPV	NPV	R^2^
APTT Actin FSL	93	67	92	78	0.59	67	81	95	30	0.53
APTT Cephascreen	100	28	85	100	0.71	85	62	93	42	0.55
PT neoplastin	93	39	86	58	0.19	98	24	88	63	0.81
PT Innovin	49	78	90	27	0.09	68	67	92	27	0.26
TT Thrombin clotin	100	33	86	100	0.18	–	–	–	–	–
TT Thrombin	100	39	86	100	0.72	–	–	–	–	–
CT-INTEM ROTEM®	52	50	86	15	0.63	77	80	95	36	0.69
CT-EXTEM ROTEM®	91	75	86	15	0.51	96	75	96	75	0.58

*PPV* Positive predictive value, *NPV* Negative predictive value, *APTT* Activated partial thrombin time, *PT* Prothrombin time, *TT* Thrombin time, *CT* Closure time

**Fig. 1:**
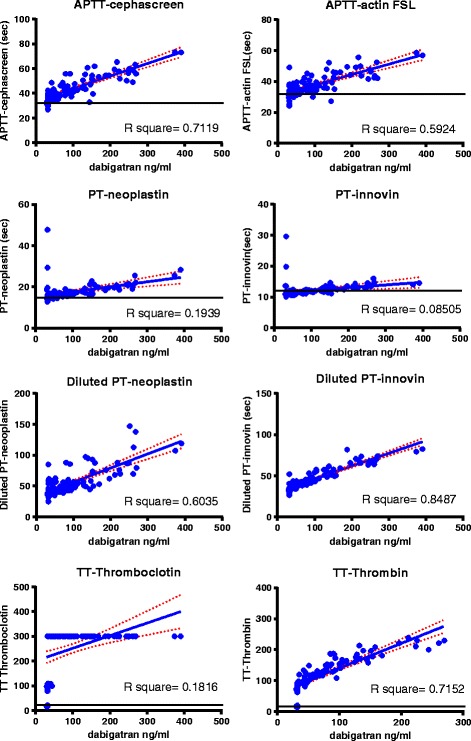
Correlations for dabigatran activity determined by diluted TT Hemoclot and the panel of coagulation assays in 75 samples from patients using dabigatran. The dotted red lines represent the standard error

APTT Cephascreen and TT Thrombin showed good linearity (R^2^ = 0.71 and R^2^ = 0.72). For the other tests linearity was moderate to poor (APTT Actin FSL (R^2^ = 0.59), PT Neoplastin (R^2^ = 0.19), Thromboclotin (R^2^ = 0.18), and PT Innovin (R^2^ = 0.09). All correlations were highly significant (all <0.0001 and 0.0046 for PT Innovin; Fig. 1, Fig. 3).

**Fig. 2:**
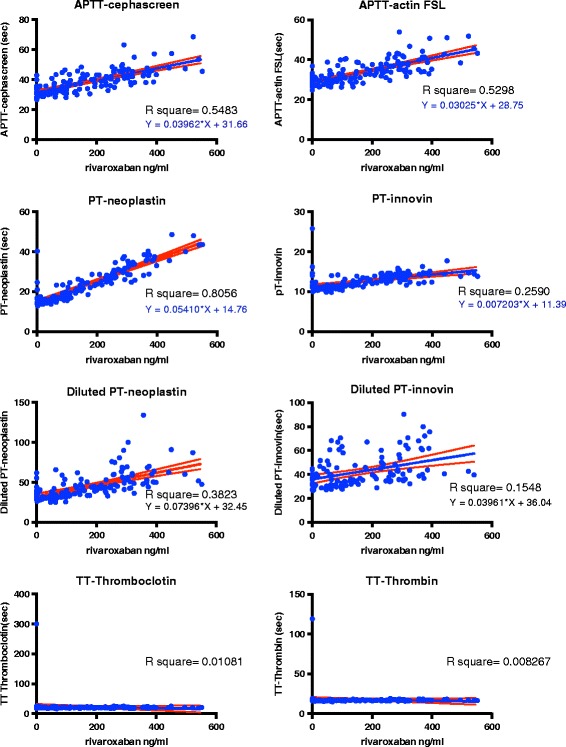
Correlations for rivaroxaban activity determined by DaXa activity for the panel of coagulationassays 109 samples from patients using rivaroxaban

**Fig. 3:**
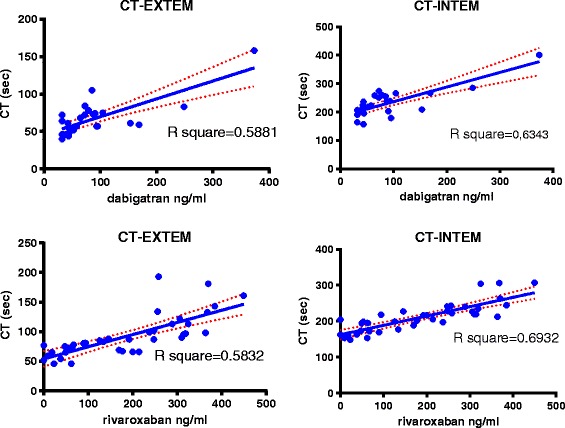
Correlations for activity levels of dabigatran (diluted TT, Hemoclot) and rivaroxaban (DaXa) withCT –EXTEM and CT-INTEM

Rivaroxaban activity was only accurately detected by two tests in the panel (PT Neoplastin (98%), and less so by APTT Cephascreen (85%). In addition Rivaroxaban activity was also accurately detected by CT intem of ROTEM® (96%). PT Innovin was not sufficiently sensitive (68%). PT Neoplastin showed good linearity across concentrations (R^2^ = 0.81). All other tests had moderate to poor linearity; APTT Cephascreen (R^2^ = 0.55), APTT Actin FSL (R^2^ = 0.53), and ROTEM® CT-EXTEM (R^2^ = 0.58). All correlations were significant (<0.0001). (Fig. 2, Fig. 3).

## Discussion

The main findings of the present study are that in patients with NVAF, the ability of routine coagulation tests to detect the presence of significant levels of dabigatran or rivaroxaban is test and reagent dependent. Therefore, emergency care protocols should ensure that local test reagents are sufficiently accurate for detecting the presence of DOACs. Otherwise, the CT-INTEM and CT-EXTEM of ROTEM® may be good and fast whole blood alternatives that may be readily available on site.

In emergency situations it is crucial that routine coagulation tests are clinically validated and that the test sensitivity for the detection of minimal and clinically important levels of anticoagulation is known. In patients with serious bleeding or requiring urgent intervention with bleeding risk, a drug concentration > 30 ng/mL is proposed as clinical relevant and sufficient to administer antidote against DOAC [9]. Emergency coagulation testing according to suggested algorithms is usually dependent on whether knowledge of the actual intake and the timing of ingestion of DOAC is available or not. If the DOAC taken by the patient is indeed known, one may choose the most appropriate test for the drug: for dabigatran the most appropriate screening test is stated to be the TT^15^. If the TT is not prolonged, the presence of dabigatran can be completely excluded. The PT is the most appropriate screening test for rivaroxaban; for most PT reagents a normal PT implies that clinical relevant concentrations of rivaroxaban can be excluded [15]. According to current guidelines, a panel of conventional coagulation tests (aPTT, PT and TT) should be used in case of intake of an unknown DOAC, to thereby exclude relevant drug levels for either dabigatran or rivaroxaban. In addition, for other DOACs such as apixaban or edoxaban, calibrated drug specific anti-Xa levels still need to be measured; screening tests are not yet available [20, 21].

However, whether the suggested algorithms are actually feasible in real life situations has not been tested yet. APTT, PT and TT are both widely and rapidly available in emergency situations, but one should be careful with the interpretation of these tests [22–25]. One needs to realize that these standard coagulation tests with low screening sensitivity might lead to a false reassurance when indicating absent drug levels. In our cohort both TT tests were able to pick up clinically relevant levels of dabigatran (≥30 ng/mL). In case of rivaroxaban however, there was a marked difference in sensitivity between the two PT tests used: PT neoplastin had a sensitivity of 97.5%, PT Innovin expressed a much lower sensitivity of only 67.7%. This may result in a underestimation of drug levels in more than 32% of patients in case PT Innovin is used for screening for active drug levels, while this is much less for PT Neoplastin (<3%). As can be appreciated from the data, most of the before mentioned tests are only useful as in- or exclusion tests (Yes, No). It would be a great advantage if also actual drug levels could be predicted form the result of a test in an emergency situation. Therefore, the correlations between drug levels and the panel of routine coagulation tests were also assessed. For dabigatran APTT Cephascreen and Thrombin were both sensitive and accurate. For rivaroxaban only PT Neoplastin was both sensitive and level predictive. Moreover, rapid measurement of DOAC plasma concentration levels have become available and might therefore also feasible for use in emergency situations [26]. Their role in the algorithms should be evaluated in the near future. For all laboratory tests applies that handling of samples by laboratory technicians is mandatory. This is time consuming and may impact swift and adequate interventions in an emergency setting. A point of care test for which whole blood can be used would improve the decision making process.

The CT-EXTEM showed good sensitivity for both rivaroxaban and dabigatran, its linearity is however moderate. Our results differ from previous studies where ROTEM® was able to show a dose dependent increase in DOAC levels in spiked whole blood samples, but poorly performed at low DOAC levels [27]. Our study showed that the ‘real life’ sensitivity of ROTEM® is better in patients using DOAC and might therefore be a good candidate for emergency testing. The added advantage of ROTEM® testing is that the results are readily available (< 5 min) and there is uniform performance with worldwide standardization. ROTEM® can be used by emergency personnel or as point of care testing in operating theaters, but also with standard situations in most hospitals laboratories. ROTEM® testing may in addition also offer the possibility of monitoring the use of antidotes.

Our study has some weaknesses: we included a relatively small number of patients and the DOACs used were limited to dabigatran and rivaroxaban, as patients in our cohort did not use apixaban or edoxaban. We furthermore did not use the accepted gold standard test, liquid chromatography with mass spectrometry (LC-MS) for quantification of DOACs [28] although the selected coagulation activity assays correlated well with LC-MS techniques.

Strengths of our study are 1) that real life samples to assess the feasibility of current guidelines on DOAC testing and 2) an extensive panel of 7 different anticoagulation tests in addition to ROTEM® testing was used.

## Conclusion

We conclude that guideline proposed screening algorithms are feasible in a real life steady state NVAF population. However, the ability of routine coagulation tests to detect the presence of significant levels of dabigatran or rivaroxaban is extremely test and reagent dependent.

Emergency care protocols should ensure that local test reagents are sufficiently accurate for detecting the presence of DOACs. CT-INTEM and CT-EXTEM of ROTEM® can be considered as effective whole blood alternatives that have the advantage to be readily available on site.

## Abbreviations

APTT: Activated Partial Thromboplastin Time
CT: Closure time
DOAC: Direct oral anticoagulants
INR: International Normalized Ratio
LC-MS: Liquid chromatography with mass spectrometry
MUMC +: Maastricht University Medical Center
NVAF: Non-valvular atrial fibrillation
PT: Prothrombin time
TT: Thrombin time
VKA: Vitamin K antagonists
VTE: Venous thromboembolism
